# C1GALT1 high expression is associated with poor survival of patients with pancreatic ductal adenocarcinoma and promotes cell invasiveness through integrin α_v_

**DOI:** 10.1038/s41388-020-01594-4

**Published:** 2021-01-08

**Authors:** Ting-Chun Kuo, Ming-Hsun Wu, Shih-Hung Yang, Syue-Ting Chen, Tzu-Wen Hsu, Jie-Yang Jhuang, Ying-Yu Liao, Yu-Wen Tien, Min-Chuan Huang

**Affiliations:** 1 Graduate Institute of Anatomy and Cell Biology, College of Medicine, National Taiwan University, Taipei, Taiwan; 2 Department of Surgery, National Taiwan University Hospital, Taipei, Taiwan; 3 Department of Traumatology, National Taiwan University Hospital, Taipei, Taiwan; 4 Department of Oncology, National Taiwan University Hospital, Taipei, Taiwan; 5 Department of Pathology, Mackay Memorial Hospital, Taipei, Taiwan

**Keywords:** Pancreatic cancer, Biomarkers

## Abstract

Pancreatic adenocarcinoma (PDAC) is a leading cause of cancer-related death. Altered glycosylation contributes to tumor progression and chemoresistance in many cancers. C1GALT1 is the key enzyme controlling the elongation of GalNAc-type O-glycosylation. Here we showed that C1GALT1 was overexpressed in 85% (107/126) of PDAC tumors compared with adjacent non-tumor tissues. High expression of C1GALT1 was associated with poor disease-free and overall survival (*n* = 99). C1GALT1 knockdown using siRNA suppressed cell viability, migration, and invasion as well as increased gemcitabine sensitivity in PDAC cells. In contrast, C1GALT1 overexpression enhanced cell migration and invasion. In subcutaneous and pancreatic orthotopic injection models, C1GALT1 knockdown decreased tumor growth and metastasis of PDAC cells in NOD/SCID mice. Mechanistically, C1GALT1 knockdown dramatically suppressed cell-extracellular matrix (ECM) adhesion, which was associated with decreased phosphorylation of FAK at Y397/Y925 and changes in O-glycans on integrins including the β_1_, α_v_, and α_5_ subunits. Using functional blocking antibodies, we identified integrin α_v_ as a critical factor in C1GALT1-mediated invasiveness of PDAC cells. In conclusion, this study not only reveals that C1GALT1 could be a potential therapeutic target for PDAC but also provides novel insights into the role of O-glycosylation in the α subunits of integrins.

## Introduction

Pancreatic ductal adenocarcinoma (PDAC) is the most common type of pancreatic cancer and characterized by an intense desmoplastic stroma, which contains mainly extracellular matrix (ECM) proteins and regulates cancer growth and metastasis [1]. Rapidly developed radio-chemoresistance, especially to the current first-line gemcitabine-based regimen, remains a critical issue underlying the poor prognosis of PDAC [2]. Abundant crosstalk between malignant epithelial cells and the surrounding desmoplastic stroma results in the proliferation, survival, and resistance to therapy of cancer cells [3]. Growing evidence has demonstrated that targeting the stroma of PDAC is a promising treatment modality in addition to surgery, chemotherapy, radiotherapy, and immunotherapy [1].

The primary cell surface receptors for various ECM proteins are integrins, which include 24 transmembrane heterodimers with 18 α and 8 β subunits [4]. Different combinations of integrins play crucial roles in nearly all steps of tumor progression, from tumorigenesis to metastasis, as well as in radio-chemoresistance [4]. Integrins are able to integrate signals from receptor tyrosine kinases (RTKs) and crosstalk with surface mucins to determine the behavior of cancer cells in the tumor microenvironment [5].

Glycosylation is the most abundant and diverse post-translational modification of proteins. Aberrant glycans have been established as biomarkers for the pathophysiology of a variety of human malignancies [6, 7], including PDAC [8]. Glycan remodeling occurs in gastrointestinal malignancies and interferes with many cellular processes, including apoptosis, growth, differentiation, invasion, immune response, migration, and transformation, which alters the ability of cancer cells to adapt to the microenvironment [6, 9]. GalNAc-type O-glycosylation is initiated by transferring N-acetylgalactosamine (GalNAc) to serine or threonine residues of proteins by 20 GalNAc transferases, namely GALNT1 to GALNT20 in humans, to form the Tn antigen [10]. C1GALT1 adds galactose (Gal) to the Tn antigen, forming the T antigen (Gal-GalNAc). To further elongate and form more complex O-glycans, other glycosyltransferases and monosaccharides are recruited. Dysregulated O-glycan biosynthesis in cancer results in truncated oligosaccharides, such as Tn, sialyl Tn, T, and sialyl T antigens [10].

Overexpression of C1GALT1 has been observed in various cancer types, including breast [11], colorectal [12], stomach [13], head and neck [14], liver [15], ovarian [16] and prostate [17] cancers. Overexpression of C1GALT1 promotes the malignant properties of these cancer cells. In contrast, C1GALT1 knockdown suppresses the malignant phenotypes. C1galt1 knockout (KO) promotes tumor development and metastasis of PDAC in mice [18]. However, the expression of C1GALT1 in PDAC and the effects of C1GALT1 overexpression or knockdown in PDAC cells remain unclear. In this study, we found that C1GALT1 is overexpressed in PDAC and high expression of C1GALT1 is associated with poor disease-free and overall survival in PDAC patients. C1GALT1 knockdown suppressed cell viability, migration, and invasion, as well as decreased tumor growth and metastasis. Conversely, C1GALT1 overexpression promoted migration and invasion. These results suggest that C1GALT1 could serve as a potential therapeutic target. Mechanistic studies showed that C1GALT1 knockdown decreased cell-ECM adhesion and integrin-FAK signaling pathways. C1GALT1 can modify O-glycans on multiple integrin subunits, and we demonstrated that integrin α_v_ plays a critical role in the C1GALT1-mediated invasiveness of PDAC cells. These findings open novel insights into the role of GalNAc-type O-glycosylation in the function of integrin α subunits and in PDAC pathogenesis.

## Results

### C1GALT1 is overexpressed and correlated with poor survival in PDAC patients

**Fig. 1:**
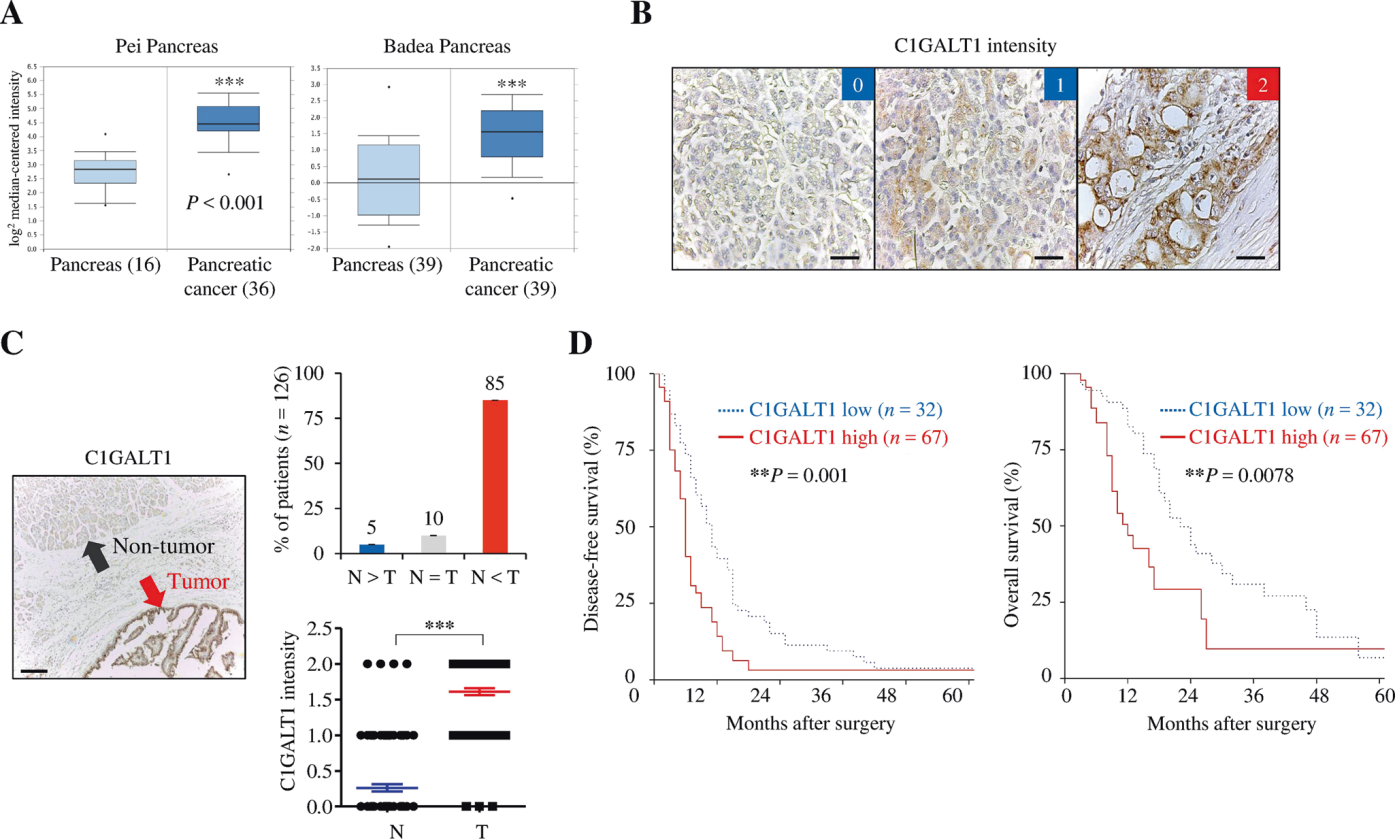
C1GALT1 is overexpressed in pancreatic cancer and is correlated with poor survival in patients. **A** *C1GALT1* mRNA levels in normal and cancerous pancreatic tissues from the Pei Pancreas (*p* = 8.08E-10) and Badea Pancreas datasets (*p* = 3.79E-8) in the Oncomine database. ***p < 0.001. **B** Intensity scoring of C1GALT1 IHC staining. C1GALT1 was immunostained using an anti-C1GALT1 monoclonal antibody. The negative control did not show any specific staining (not shown). Scale bars, 50 μm. **C** Representative images showing C1GALT1 in pancreatic ductal adenocarcinoma (red arrow) and adjacent non-tumor tissue (black arrow). Scale bars, 400 μm. Statistical analyses of C1GALT1 expression in paired pancreatic tumors. N non-tumor. T pancreatic ductal adenocarcinoma. *n* = 126. ***p < 0.001. **D** Kaplan–Meier plots of disease-free and overall survival. C1GALT1 scores 0 and 1 and score 2 were classified as low (*n* = 32) and high (*n* = 67) expression, respectively. **p < 0.01 by log rank test.

Using data from the Oncomine database, *C1GALT1* mRNA levels are found to be significantly increased in pancreatic cancer compared with normal pancreatic tissues in both the Pei and Badea Pancreas datasets (Fig. 1A). To investigate C1GALT1 protein expression in paired pancreatic adenocarcinomas (PDACs) and their adjacent non-tumor tissues, we collected clinical samples from a tissue microarray (*n* = 27) (Biomax PA811) and the National Taiwan University Hospital (*n* = 99) (Taiwan). C1GALT1 was immunostained using an anti-C1GALT1 mouse monoclonal antibody and the staining intensity of C1GALT1 was scored from 0 to 2 (Fig. 1B). The results indicated that 85% (107/126) of PDAC tumor tissues had higher C1GALT1 levels than their corresponding non-tumor tissues (T > N) (Fig. 1C). In sharp contrast, only about 5% (6/126) of PDAC tumor tissues showed lower C1GALT1 levels (T < N). Among the 126 tested PDAC tumors, only three did not express C1GALT1 at detectable levels under our experimental conditions. A paired student’s *t* test indicated that C1GALT1 was significantly overexpressed in PDAC compared with adjacent non-tumor tissue (Fig. 1C). Because there was no survival information in the tissue microarray tested, we used 99 patients from the National Taiwan University Hospital for survival analysis. For further statistical analysis, C1GALT1 levels in PDAC tissues were classified as either low (scores 0 and 1) or high (score 2) expression. Kaplan–Meier survival analysis showed that high C1GALT1 expression correlated with poor disease-free and overall survival in PDAC patients (Fig. 1D). These results suggest that C1GALT1 is overexpressed in PDAC and high expression of C1GALT1 predicts poor survival in PDAC patients.

### C1GALT1 knockdown inhibits whereas C1GALT1 overexpression enhances malignant behaviors in PDAC cells

**Fig. 2:**
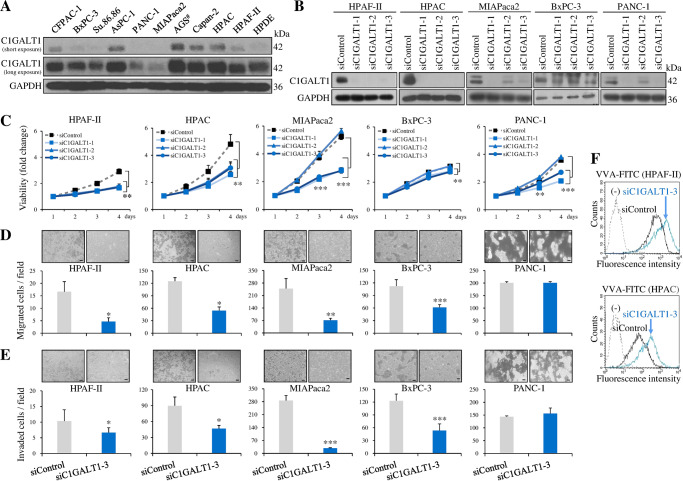
C1GALT1 knockdown inhibits malignant behaviors in pancreatic cancer cells. **A** Western blots showing C1GALT1 expression in nine pancreatic cancer cell lines, one non-transformed human pancreatic duct epithelial (HPDE) cell, and one gastric cancer cell line AGS (#), as indicated. Blots with short and long exposure times are shown. GAPDH was used as an internal loading control. **B** Western blots showing C1GALT1 transient knockdown in multiple pancreatic cancer cells. C1GALT1 was knocked down using three independent siRNAs (siC1GALT1-1, siC1GALT1-2, and siC1GALT1-3) in HPAF-II, HPAC, MIAPaca2, BxPC-3, and PANC-1 cells. Non-targeting siRNA (siControl) was used as control. GAPDH was used as an internal loading control. **C** C1GALT1 knockdown inhibited cell viability. Cell viability was analyzed using 4-day MTT assays at different time points, as indicated, in HPAF-II, HPAC, MIAPaca2, BxPC-3, and PANC-1 cells. Results are presented as mean ± SD of three independent experiments using student’s *t* test. **p < 0.01 and ***p < 0.001 by student’s *t* test. **D** C1GALT1 knockdown inhibited cell migration. Cell migration was analyzed using Transwell migration assays for 24 h in HPAF-II, HPAC, MIAPaca2, BxPC-3, and PANC-1 cells. Results are presented as mean ± SD of three independent experiments. Representative images of migrated cells are shown. Scale bars, 100 μm. *p < 0.05, **p < 0.01, and ***p < 0.001 by student’s *t* test. **E** C1GALT1 knockdown inhibited invasion. Cell invasion was analyzed using Matrigel invasion assays for 24 h in HPAF-II, HPAC, MIAPaca2, BxPC-3, and PANC-1 cells. Results are presented as mean ± SD of three independent experiments. Representative images of invaded cells are shown. Scale bars, 100 μm. *p < 0.05 and ***p < 0.001 by student’s *t* test. **F** Representative flow cytometric histograms showing the effects of C1GALT1 knockdown on the expression of cell surface Tn antigens. C1GALT1 was knocked down using siC1GALT1-3 in HPAF-II and HPAC cells. Tn antigens were detected by VVA lectin conjugated with FITC. Unstained cells were used as a negative control (-).

To investigate the potential role of C1GALT1 in PDAC, C1GALT1 was knocked down or overexpressed in PDAC cells and then MTT viability, transwell migration, and Matrigel invasion assays were performed. Western blot analysis revealed variable C1GALT1 expression levels in 9 PDAC cells and one non-transformed human pancreatic ductal epithelial (HPDE) cell (Fig. 2A). HAPF-II, HPAC, MIAPaca2, BxPC-3, and PANC-1 cells were subjected to C1GALT1 knockdown using three independent C1GALT1-specific siRNAs and two independent C1GALT1-specific shRNA constructs to overcome potential off-target effects. All three siRNAs, except for siC1GALT1-2 (Fig. 2B), and two shRNAs (Supplementary Fig. S1A) demonstrated a high efficiency of C1GALT1 knockdown. Our results showed that C1GALT1 knockdown significantly suppressed viability, migration, and invasion in HPAF-II, HPAC, MIAPaca2, and BxPC-3, but not in PANC-1 cells (Fig. 2C–E; Supplementary Fig. S1B). Flow cytometry with VVA lectin showed that C1GALT1 knockdown increased Tn antigens on cell surfaces in HAPF-II and HPAC cells (Fig. 2F), indicating that O-glycan extension is reduced to form more short O-glycans. Next, we examined whether itraconazole, a C1GALT1 inhibitor described in our previous report [14], could decrease cell viability in PDAC and non-transformed HPDE cells. Our data showed that itraconazole treatment decreased C1GALT1 levels and increased Tn antigens on cell surfaces in HPAC cells (Supplementary Fig. S2A), confirming that itraconazole is also a C1GALT1 inhibitor in pancreatic cancer cells. Moreover, treatment of cells with 2 µM itraconazole for 72 h decreased cell viability of HPAF-II, HPAC, and HPDE cells to 69.5%, 34.1%, and 75.0%, respectively (Supplementary Fig. S2B), implying that PDAC cells could be more sensitive to C1GALT1-targeted drugs than non-transformed HPDE cells.

To further confirm the effects of C1GALT1 in PDAC cells, C1GALT1 was overexpressed in MIAPaca2 and HPAF-II cells using C1GALT1/pcDNA3.1 plasmid. The results revealed that C1GALT1 overexpression significantly enhanced PDAC cell migration and invasion (Supplementary Fig. S3). However, C1GALT1 overexpression did not significantly affect cell viability in MIAPaca2 and HPAF-II cells probably due to weak overexpression. These results suggest that C1GALT1 knockdown suppresses cell viability, migration, and invasion in PDAC cells. In contrast, C1GALT1 overexpression increases cell migration and invasion in PDAC cells.

### C1GALT1 knockdown increases sensitivity to gemcitabine in PDAC cells

**Fig. 3:**
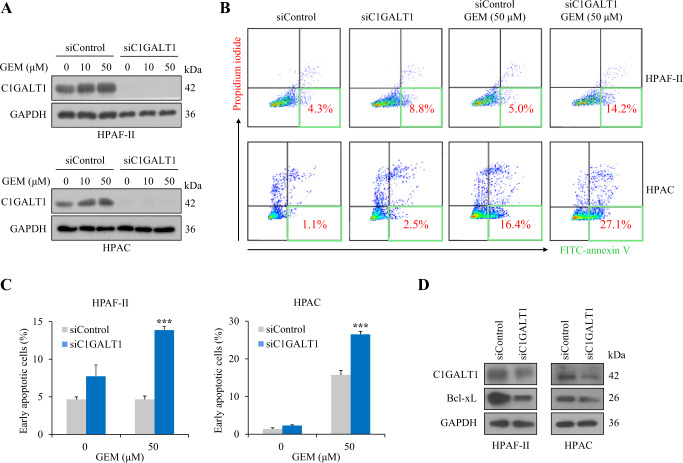
C1GALT1 knockdown increases gemcitabine sensitivity in pancreatic cancer cells. **A** Western blots showing transient knockdown of C1GALT1 in HPAF-II and HPAC cells treated with gemcitabine (GEM) at different concentrations, as indicated, for 24 h. GAPDH was used as an internal loading control. **B** Flow cytometric analysis with FITC-annexin V and propidium iodide. C1GALT1 was knocked down using siC1GALT1-3 in HPAF-II and HPAC cells treated with/without 50 µM gemcitabine (GEM), as indicated. Representative flow cytometric data are shown. Numbers in the green rectangles indicate the percentage of early apoptotic cells. **C** Quantification of early apoptotic cells from (**B**). Results are presented as mean ± SD of three independent experiments. ***p < 0.001 by student’s *t* test. **D** Western blots showing the effects of C1GALT1 knockdown on B-cell lymphoma-extra large (Bcl-xL). GAPDH was used as an internal loading control.

Gemcitabine is a standard chemotherapeutic drug used to treat PDAC. Unfortunately, gemcitabine resistance is developed in most treated patients. Given that silencing of C1GALT1 reduced cell viability, we next investigated whether C1GALT1 knockdown could overcome gemcitabine resistance in PDAC cells. To address this, C1GALT1 was knocked down and cell death was analyzed by flow cytometry or immunofluorescence using FITC-annexin V and propidium iodide. Transient knockdown of C1GALT1 with siRNA (Fig. 3A) or stable knockdown of C1GALT1 with lentivirus-mediated shRNA (Supplementary Fig. S4) in HPAF-II and HPAC cells treated with gemcitabine was confirmed by Western blotting. Flow cytometry revealed that, in gemcitabine-treated cells, the percentage of early apoptotic cells was significantly increased upon siRNA-mediated C1GALT1 knockdown in both HPAF-II and HPAC cells (Fig. 3B, C). In HPAF-II and HPAC cells with stable knockdown of C1GALT1, gemcitabine-induced apoptotic cells were also increased compared with control knockdown cells (Supplementary Fig. S4). In line with this finding, among apoptosis-related molecules tested, C1GALT1 knockdown decreased the expression of the anti-apoptotic factor Bcl-xL in HPAF-II and HPAC cells (Fig. 3D).

### C1GALT1 knockdown inhibits tumor growth and metastasis in vivo

**Fig. 4:**
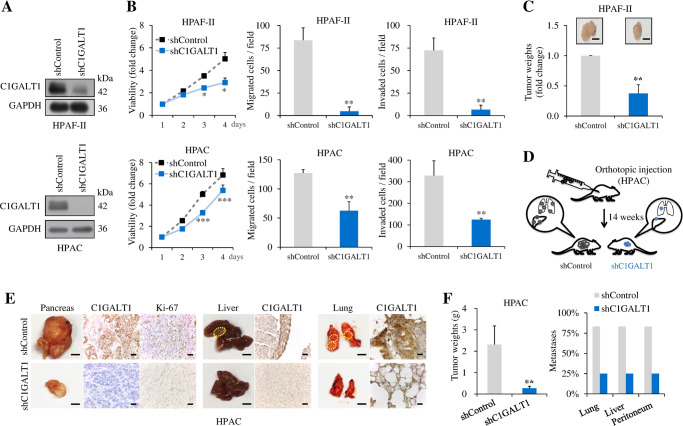
C1GALT1 knockdown inhibits tumor growth and metastasis in NOD/SCID mice. **A** Western blots showing stable knockdown of C1GALT1 in HPAF-II and HPAC cells. C1GALT1 was knocked down using lentivirus-mediated C1GALT1 shRNA in pLKO.1 vector (shC1GALT1). Non-targeting shRNA in pLKO.1 vector (shControl) was used as a control. GAPDH was used as an internal loading control. **B** Effects of C1GALT1 knockdown on cell viability, migration, and invasion were analyzed using 4-day MTT, Transwell migration, and Matrigel invasion assays, respectively. Results are presented as mean ± SD of three independent experiments. *p < 0.05, **p < 0.01, and ***p < 0.001 by student’s *t* test. **C** Effects of C1GALT1 knockdown on tumor growth. Representative images of tumors excised from NOD/SCID mice 17 weeks after subcutaneous injection with HPAF-II cells are shown. Scale bars, 10 mm. Tumors were weighed and quantified as fold change. *n* = 5 for each group. **p < 0.01. **D** Schematic diagram illustrating how experiments for tumor growth and distant metastasis assays in vivo were performed. HPAC cells (5 × 10^6^) and immortalized pancreatic stellate cells in a 1:1 ratio were injected orthotopically into NOD/SCID mice. After 14 weeks, pancreatic tumors, livers, and lungs were excised for analysis. **E** Effects of C1GALT1 knockdown on tumor growth and metastasis. Representative images of the tissues excised from mice injected orthotopically with HPAC cells are shown. The excised tissues were paraffin-embedded and immunostained for C1GALT1 and Ki-67, as indicated. Yellow dotted circles indicate metastatic tumors. Scale bars, 5 mm. **F** Statistical results of tumor weights and metastases. Tumors grown in the pancreas were weighed. Results are presented as mean ± SD. **p < 0.01 by paired student’s *t* test. Metastases of the lung, liver, and peritoneum were assessed. Results are presented as percentage. *n* = 6 and 4 are for shControl and shC1GALT1 group, respectively.

To further demonstrate the role of C1GALT1 in vivo, control and C1GALT1 stable knockdown PDAC cells were subcutaneously or orthotopically injected into NOD/SCID mice and tumor growth and metastasis were analyzed. First, C1GALT1 was stably knocked down using lentivirus-mediated non-targeting shRNAs (shControl) or C1GALT1 shRNA (shC1GALT1) in HPAF-II or HPAC cells (Fig. 4A). Consistent with the previous results, C1GALT1 stable knockdown significantly suppressed cell viability, migration and invasion in vitro in both HPAF-II and HPAC cells (Fig. 4B). In the subcutaneous injection model, C1GALT1 knockdown significantly decreased the tumor weights of HPAF-II cells (Fig. 4C). In the orthotopic injection model, 14 weeks after HPAC cells were injected into the pancreas of mice, C1GALT1 knockdown decreased the tumor weights in the pancreas and inhibited metastases to the liver, lungs, and peritoneum (Fig. 4D–F). These results suggest that C1GALT1 knockdown inhibits tumor growth and metastasis of PDAC cells in NOD/SCID mice.

### C1GALT1 knockdown suppresses cell-ECM adhesion and integrin-FAK signaling as well as alters O-glycans on integrins in PDAC cells

**Fig. 5:**
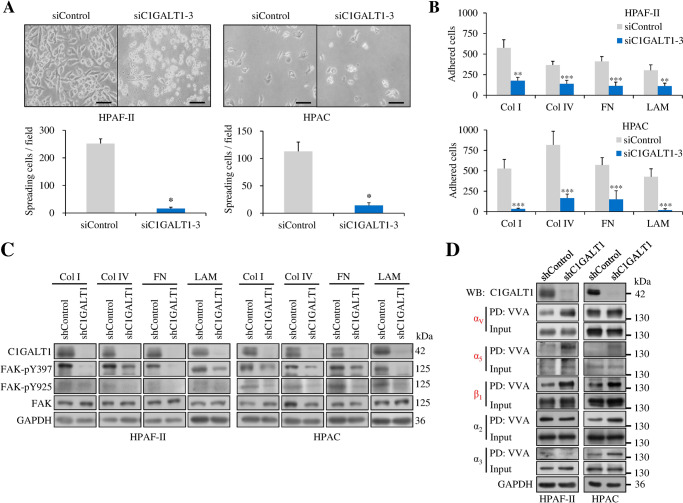
C1GALT1 modulates cell adhesion, integrin-FAK signaling, and integrin O-glycosylation in pancreatic cancer cells. **A** Effects of C1GALT1 knockdown on cell spreading in HAPF-II and HPAC cells. Pancreatic cancer cells were treated with control (siControl) or C1GALT1 siRNA (siC1GALT1-3). Representative images showing amplified cells in one fourth of a field. Scale bars, 20 μm. Spreading cells were quantified under a phase-contrast microscope and are shown in the lower panel. *p < 0.05 by student’s *t* test. **B** C1GALT1 knockdown inhibited cell-extracellular matrix (ECM) adhesion. Cells were plated onto 96-well plates coated with 2.5 μg/μL of bovine serum albumin (BSA), collagen I (Col I), collagen IV (Col IV), fibronectin (FN), or laminin (LAM). Specific ECM-adhered cells were calculated by subtracting BSA-adhered cells. Results are presented as mean ± SD of six independent experiments. **p < 0.01; ***p < 0.001 by student’s *t* test. **C** Effects of C1GALT1 knockdown on tyrosine phosphorylation of FAK. HPAF-II and HPAC cells were plated onto culture plates coated with 1 µg/mL of different ECM proteins, as indicated, in serum-free DMEM for 3 h. Changes in FAK phosphorylation at Y397 and Y925 were analyzed by Western blotting. GAPDH was used as an internal loading control. **D** Effects of C1GALT1 knockdown on Tn antigen expression of selected integrins, including α_v_, α_5_, β_1_, α_2_, and α_3._ Changes in Tn expression were analyzed using VVA pull-down (PD) assays in C1GALT1 knockdown HAPF-II and HPAC cells. Proteins were detected by Western blot (WB) analysis. GAPDH was used as an internal loading control.

To investigate the mechanisms by which C1GALT1 regulates the malignant behaviors of PDAC cells, we next analyzed RTKs and integrins given that they are critical surface glycoprotein receptors for cell growth and metastasis [12–15, 19]. First, we knocked down C1GALT1 using siRNA and analyzed changes in the levels of 49 phospho (p)-RTKs using p-RTK arrays. Consistent with previous reports [13, 14, 19], our data showed that C1GALT1 knockdown decreased the levels of multiple p-RTKs, including p-EGFR, p-ErbB2, p-MET, and p-MSPR (Supplementary Fig. S5), although the changes were mild. Interestingly, in HPAF-II and HPAC cells, we consistently found that C1GALT1 knockdown dramatically suppressed cell spreading on the substratum (Fig. 5A). At the molecular level, although E-cadherin was only marginally affected by C1GALT1 siRNA, N-cadherin levels were decreased (Supplementary Fig. S6). Several lines of evidence indicate that cancer cells exhibit both epithelial and mesenchymal characteristics, that is known as partial epithelial mesenchymal transition (EMT), during cancer progression and partial EMT can promote cancer invasiveness [20]. Our results therefore suggest that decrease in C1GALT1 represses EMT in PDAC cells. Based on the above findings, we hypothesized that C1GALT1 could modulate integrin signaling pathways and in turn regulate PDAC cell invasiveness. Indeed, cell adhesion assays revealed that C1GALT1 knockdown significantly suppressed cell adhesion to multiple ECMs, including collagen I, collagen IV, fibronectin, and laminin, in HPAF-II and HPAC cells (Fig. 5B). To further confirm this effect at the molecular level, we analyzed the phosphorylation of FAK, the most important downstream signaling molecule of integrins [21, 22], in control and C1GALT1 knockdown PDAC cells seeded onto plates coated with the above ECM proteins. Our results showed that C1GALT1 knockdown inhibited phosphorylation of FAK at Y397 and Y925 in all tested ECMs in both HPAF-II and HPAC cells (Fig. 5C). Although β_1_ integrin has been reported to carry O-glycans [15], it is largely unknown whether the α subunits of integrins express O-glycans. As expected, VVA lectin pull-down assays showed that C1GALT1 knockdown increased VVA binding to β_1_ integrin (Fig. 5D). Interestingly, we also found that C1GALT1 knockdown increased the Tn antigen on integrins α_v_ and α_5_ in both HPAF-II and HPAC cells (Fig. 5D). In contrast, the effect of C1GALT1 knockdown on VVA binding to integrins α_2_ and α_3_ was not consistent in these two cell lines. Furthermore, the C1GALT1 expression level is inversely correlated with the Tn antigen level on integrins α_V_ and α_5_ in HPAC, HPAF-II, BxPC-3, and MIAPaca2 cells (Supplementary Fig. S7). These results suggest that C1GALT1 knockdown not only inhibits cell-ECM adhesion and integrin-FAK signaling but also alters O-glycans on integrins in PDAC cells.

Next, we examined whether the integrin-FAK signaling plays a role in gemcitabine response. We inhibited FAK functions using FAK inhibitor 14 (sc-203950) and assessed its effect on gemcitabine-induced cell death by MTT assays. Our data showed that FAK inhibition significantly increased gemcitabine-induced cell death (Supplementary Fig. S8), which phenocopies the effect of C1GALT1 knockdown. These results suggest that C1GALT1 knockdown sensitizes PDAC cells to gemcitabine and the integrin-FAK signaling pathway is involved in the underlying mechanism.

### Integrin αv is involved in C1GALT1-mediated invasion in PDAC cells

**Fig. 6:**
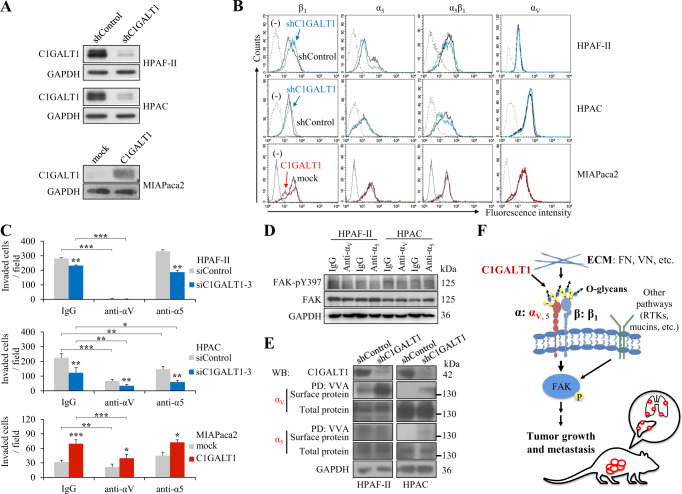
Integrin α_v_ is involved in C1GALT1-mediated invasion in pancreatic cancer cells. **A** Western blots showing C1GALT1 stable knockdown in HPAF-II and HPAC cells and C1GALT1 overexpression in MIAPaca2 cells. PDAC cells were transfected using lentivirus-mediated C1GALT1 shRNA in pLKO.1 vector (shC1GALT1) compared with its empty vector (shControl) and C1GALT1 was overexpressed using C1GALT1/pcDNA3.1 plasmid (C1GALT1) compared with its empty plasmid (mock). GAPDH was used as an internal loading control. **B** C1GALT1 knockdown or overexpression did not alter the expression of surface integrins β_1_, α_5_, α_5_β_1_, and α_v_ in HAPF-II and HPAC cells analyzed by flow cytometry. Unstained cells were used as a negative control (-). **C** Effects of functional blocking antibodies against integrins on PDAC cell invasion. C1GALT1 knockdown HPAF-II and HPAC cells and C1GALT1 overexpressing MIAPaca2 cells were subjected to Matrigel invasion assays. Cells were treated with 10 μg/mL of blocking antibody, as indicated. IgG was used as a control. Invasion of HPAF-II, HPAC, and MIAPaca2 cells was analyzed after 24 and 48 h. Results are presented as mean ± SD of four independent experiments. *p < 0.05; **p < 0.01; ***p < 0.001 by student’s *t* test. **D** Effects of functional blocking antibodies on FAK phosphorylation in PDAC cells using Western blotting. Functional blocking antibody against integrin α_v_ or α_5_ was used, as indicated, to treat HPAF-II and HPAC cells for 30 min before seeding to culture plates. IgG was used as a control. GAPDH was used as an internal loading control. **E** Western blots showing changes in Tn antigens on cell surface integrins α_V_ and α_5_. Cells were surface biotinylated. Plasma Membrane Protein Extraction Kit (Abcam) was used for extraction and purification of plasma membrane proteins from HPAF-II and HPAC cells. VVA pull-down (PD) assays were performed to assess changes in Tn antigens on integrins. Proteins were detected by Western blot (WB) analysis. **F** A schematic diagram illustrating the proposed mechanism by which C1GALT1 promotes tumor growth and metastasis in pancreatic cancer. C1GALT1 modifies O-glycans on integrins, including α_5_, α_v_, and β_1_, which leads to altered integrin-FAK signaling. Integrin α_v_ (red color) is proposed to play a critical role in C1GALT1-mediated invasiveness. This pathway is coordinated with other C1GALT1-regulated pathways, such as receptor tyrosine kinases (RTKs) and mucins, to promote tumor growth and metastasis in pancreatic cancer. FN fibronectin, VN vitronectin.

In addition to the well-studied integrin β_1_, our data revealed that C1GALT1 was able to consistently modulate O-glycosylation of integrins α_5_ and α_v_. This prompted us to further investigate the role of integrins α_5_ and α_v_ in C1GALT1-mediated invasiveness of PDAC cells. C1GALT1 knockdown in HPAF-II and HPAC cells or overexpression in MIAPaca2 cells was confirmed by Western blotting (Fig. 6A). Flow cytometry indicated that C1GALT1 knockdown did not significantly affect surface levels of integrin α_5_ and α_v_ in these three cell lines (Fig. 6B). In addition, surface levels of integrin α_5_β_1_ and β_1_ were also not significantly changed. Using a functional blocking antibody, we found that blocking α_v_ functions significantly inhibited cell invasion in HPAF-II, HPAC, and MIAPcaca2 cells, as well as significantly decreased the C1GALT1-mediated invasion in MIAPcaca2 cells (Fig. 6C). In contrast, blocking integrin α_5_ functions suppressed cell invasion in HPAC cells, but not in HPAF-II and MIAPaca2 cells. Our results consistently showed that treatment with an integrin α_v_ blocking antibody decreased FAK phosphorylation although the change was not dramatic (Fig. 6D). To further demonstrate the role of integrin α_v_ in FAK phosphorylation in PDAC cells, we analyzed FAK-pY397 levels in PDAC cells treated with cilengitide, a specific inhibitor of α_v_-containing integrins. Our results showed that cilengitide significantly decreased phospho-FAK levels in both HPAF-II and HPAC cells (Supplementary Fig. S9), indicating that integrin α_v_ indeed plays a critical role in FAK phosphorylation in PDAC cells. To examine whether the O-glycosylation of cell surface integrins α_v_ and α_5_ was modulated by C1GALT1, cells were surface biotinylated and then analyzed by VVA pull-down assays. The results showed that C1GALT1 knockdown consistently increased Tn antigens on integrin α_v_ in both HPAF-II and HPAC cells (Fig. 6E), indicating that C1GALT1 can modulate O-glycosylation of cell surface integrin α_v_ in PDAC cells. Given that, these results suggest that integrin α_v_ subunit plays a critical role in the C1GALT1-mediated invasion in PDAC cells.

## Discussion

In this study, we showed that 85% of PDAC tumors exhibited higher C1GALT1 expression levels than their adjacent non-tumor pancreatic tissues. High expression of C1GALT1 is associated with poor disease-free and overall survival. C1GALT1 knockdown suppressed tumor growth and invasiveness in vitro and in vivo. In vitro studies revealed that C1GALT1 knockdown dramatically decreased cell-ECM adhesion and inhibited ECM-triggered tyrosine phosphorylation of FAK, a critical downstream signaling molecule of integrins and associated with invasiveness of multiple caners [5, 22, 23]. In addition to integrin β_1_, which has repeatedly been demonstrated to be O-glycosylated and regulated by C1GALT1 in several cancers, we found that the α subunits of integrins, including α_v_ and α_5_, were also modified by C1GALT1. Using a functional blocking antibody, we found that integrin α_v_ is involved in C1GALT1-mediated invasion of PDAC cells. This study provides novel insights into the role of C1GALT1-mediated O-glycosylation in PDAC pathogenesis and demonstrates that integrin α_v_ plays a critical role in the C1GALT1-mediated invasiveness of PDAC cells.

The most common β subunit in integrin heterodimers is β_1_, which can partner with any one of 11 α subunits (α_1_ – α_11_) to mediate cell adhesion to various ECM proteins [24]. We and others have demonstrated that O-glycans on integrin β_1_ are modified by C1GALT1 and can regulate cell-ECM adhesion [15, 25]. Here, in support of this finding, we showed that silencing of C1GALT1 altered the O-glycans of integrin β_1_ and decreased cell adhesion to various ECM proteins, which was associated with decreased tyrosine phosphorylation of FAK at Y397 in PDAC cells. However, the functional role of O-glycans on integrin α subunits has long been ignored. Among the α subunits, α_v_ can associate with several β subunits, including β_1_, β_3_, β_5_, β_6_, and β_8_ [26]. Proteins containing RGD peptides are their major ligands, such as fibronectin and vitronectin. The α_v_-containing integrin heterodimers have been reported to control multiple functions of tumors, including tumor growth, metastasis, angiogenesis, immunomodulation, and bone metastasis [27]. Several small molecules and therapeutic antibodies against integrin α_v_ have been developed for cancer treatment and are listed in clinical trials [26]. Here, for the first time, we demonstrated that integrin α_v_ was decorated with Tn antigens and its O-glycans were modulated by C1GALT1. Furthermore, antibody-mediated blockade of integrin α_v_ not only suppressed PDAC cell invasion, but also significantly decreased C1GALT1-mediated invasion. Our results strongly suggest that integrin α_v_ carries O-glycans and the O-glycosylation can modulate functions of integrin α_v_. In contrast, although our data demonstrated that integrin α_5_ expressed Tn antigens that could be modified by C1GALT1 in HPAF-II cells, blocking integrin α_5_ did not significantly affect cell invasion. The effect of C1GALT1-mediated O-glycosylation on integrin α_5_ in PDAC cells requires further investigation. These findings suggest that GalNAc-type O-glycosylation could be a common modification in the α subunits of integrins. To thoroughly understand integrin functions, it will be of great importance to study the role of O-glycans in the interaction between integrin α and β subunits.

The proposed mechanism by which C1GALT1 promotes tumor growth and metastasis in PDAC is illustrated in Fig. 6F. Briefly, C1GALT1 modifies O-glycans on integrins, especially the α_V_, α_5_, and β_1_ subunits, and increases the integrin-FAK signaling to promote cell invasiveness. Previous reports and this study have shown that C1GALT1 is able to increase the activity of multiple important RTKs [12–14, 19]. Moreover, our previous study demonstrated that mucin (MUC) 20, a highly O-glycosylated protein, promotes PDAC tumor growth and metastasis [28]. Therefore, we proposed that the integrin-mediated pathway is coordinated with other C1GALT1-regulated pathways, including RTKs and mucins, to promote tumor growth and metastasis in pancreatic cancer. This study demonstrates that knockdown of C1GALT1 is sufficient to suppress tumor growth and invasiveness in vitro and in vivo, implying that C1GALT1 is a potential therapeutic target for PDAC treatment.

Chugh et al. reported that knockout of *C1galt1* promoted development and metastasis of PDAC in mice [18]. However, our data showed that knockdown of *C1GALT1* in human PDAC cells suppressed tumor growth and metastasis in NOD/SCID mouse xenograft models. These phenotypic differences between knockouts and knockdowns in a number of model systems, including zebrafish, mouse, and human cell lines, indeed have been observed in many studies [29]. To rule out the possibility of off-target effects, we used several independent siRNAs and shRNAs to knockdown *C1GALT1* in multiple PDAC cells and obtained similar phenotypic effects both in vitro and in vivo. Moreover, genetic compensation in response to gene knockout has been recently reported to be a widespread phenomenon [29]. Therefore, it is very likely that the effect of *C1galt1* knockout is attributed by genetic compensation. We found that 97.6% (123/126) of PDAC tumors expressed C1GALT1 at detectable levels by immunohistochemistry. In contrast to the knockout system that causes a complete loss of C1GALT1 expression in PDAC cells, we used siRNA-mediated knockdown to mimic low levels of C1GALT1 in PDAC. Consistent with clinical analysis indicating that lower C1GALT1 expression is associated with better overall survival in PDAC patients, reducing C1GALT1 expression using siRNA suppressed tumor growth and metastasis of PDAC cells. These findings also imply that when C1GALT1 is used as a therapeutic target, decreases in C1GALT1 activity will be beneficial for PDAC patients. Notably, a complete loss of the *C1GALT1* gene or C1GALT1 enzymatic activity may be harmful to patients with PDAC and should be avoided.

## Materials and methods

### Clinical samples

Pancreatic cancer tissue microarray (Biomax PA811) with matched cancer adjacent tissues was purchased from US Biomax, Inc. (Rockville, MD, USA). Tissue slides of 99 PDAC patients were obtained from National Taiwan University Hospital with IRB approval (201411085RINB). The information of PDAC patients is listed in Supplementary Table S1.

### Immunohistochemical (IHC) staining

The protein expression of C1GALT1 was recognized with anti-C1GALT1 mouse monoclonal antibody (Santa Cruz Biotechnology, Inc., CA, USA) and was detected by UltraVision Quanto Detection System (Thermo Scientific, Cheshire, UK). Pancreatic cancer tissue microarray (Biomax PA811, US Biomax, Inc., Rockville, MD, USA) containing 27 cases and paraffin-embedded blocks of tumors from 99 surgical human tissue samples from National Taiwan University Hospital were immunostained for C1GALT1. The IHC staining assessment was independently conducted by two pathologists who were blinded to patient outcomes.

### Cell lines and cell culture

PDAC cell lines, including CFPAC-1, BxPC-3, Su.86.86, AsPC-1, PANC-1, MIA PaCa2, Capan-2, HPAC, and HPAF-II were generously provided by Dr WH Lee (Genomics Research Center, Academia Sinica, Taipei, Taiwan) and were confirmed using Short Tandem Repeat (STR) DNA profiling. The non-transformed HPDE cells generated from a 75-years old male pancreatic specimen (a gift from Dr Kelvin K. Tsai, National Health Research Institutes, Taiwan) were grown in keratinocyte serum-free (KSF) medium with 0.2 ng/ml EGF and 30 μg/ml bovine pituitary extract (Invitrogen Life Technologies). One immortalized pancreatic stellate cell (HPaSteC) line was purchased from ScienCell Research Laboratories, inc (Carlsbad, CA). PANC-1 and MIAPaCa2 cells were maintained in Dulbecco’s modified Eagle’s medium (DMEM) (Thermo Fisher Scientific, Grand Island, NY, USA). CFPAC-1, Capan-2, HPAC, HPAF-II, and HPaSteC cells were maintained in DMEM/F-12 GlutaMAXTM (Invitrogen^™^). BxPC-3, Su.86.86, and AsPC-1 cells were maintained in Roswell Park Memorial Institute (RPMI) 1640 medium (Thermo Fisher Scientific). The medium was supplemented with 10% Fetal Bovine Serum (FBS) (Thermo Fisher Scientific), 100 IU/mL penicillin, and 100 μg/mL streptomycin (Thermo Fisher Scientific) and cells were cultured at 37 °C with 5% CO_2_ in air.

### Transfection and plasmid constructs

C1GALT1 was transiently knocked down using three independent C1GALT1-specific siRNAs (Invitrogen^™^) and two C1GALT1-specific shRNA constructs in pLKO.1 and pSUPER.retro vectors. Non-targeting siRNA (Invitrogen^™^) and shRNA were used as a control. Cells were transiently transfected for 24–48 h with 10 nM of siRNA using Lipofectamine RNAiMAX (Invitrogen^™^) or with 10 nM of shRNA by Lipofectamine 3000 (Invitrogen^™^). The siRNAs against C1GALT1 were si-C1GALT1-1: 5′-UUAGUAUACGUUCAGGUAAGGUAGG-3′, si-C1GALT1-2: 5′-UUAUGUUGGCUAGAAUCUGCAUUGA-3′, and si-C1GALT1-3: 5′-CCUACCUUACCUGAACGUAUACUAA-3′. The non-targeting siRNA (si-Control) was 5′-CAACCUCAGCCAUGUCGACUGGUUU-3′. Stable knockdown was performed using shRNA constructs in pLKO.1 vector (TRCN35411: 5′-CCGGCCCAGCCTAATGTTCTTCATACTCGAGTATGAAGAACATTAGGCTGGGTTTTTG-3′ from RNAi Core, Academia Sinica, Taipei, Taiwan) through lentivirus-based transfection system. Non-targeting shRNA (TRC025 from RNAi Core, Academia Sinica, Taipei, Taiwan) was used as a control. Stable knockdown clones were selected using 1 μg/ml of puromycin (Sigma, St. Louis, MO, USA). C1GALT1 overexpressing cells and the corresponding control cells were obtained using C1GALT1/pcDNA3.1 plasmid or empty vector pcDNA3.1 (RNAi Core, Academia Sinica, Taipei, Taiwan) through lipofectamine 3000 transfection system and 1 mg/ml of Geneticin (G418, Gibco^®^, Thermo Fisher Scientific) was used to select stable clones.

### Antibodies and reagents

Antibodies against C1GALT1(F-31), FAK (C-20), and integrin α_5_ (H-104) were obtained from Santa Cruz Biotechnology, Inc. (Santa Cruz, CA, USA). Antibodies against Bcl-xL (54H6) and phospho-FAK (Tyr397 and Tyr925) were purchased from Cell Signaling Technology, Inc. (Danvers, MA, USA). Ki-67 was obtained from Vector Laboratories (Burlingame, CA, USA). CD-31 (PECAM-1) and integrin β1 (CD29) were purchased from BD Pharmingen (San Jose, CA, USA). Antibody against GAPDH (0411) was purchased from GeneTex, Inc. (Irvine, CA, USA). Antibodies against integrin α_v_ (P3G8), integrin α_2_ (CD49b), and integrin α_3_ (CD49c) were obtained from Merck KGaA (Darmstadt, Germany). Functional blocking antibodies for integrin β_1_ (P4C10), integrin α_5_β_1_ (JBS5), integrin α_5_ (P1D6), and integrin α_v_ (AV1) were purchased from Merck KGaA. Gemzar (gemcitabine HCl) was obtained from Lilly Medical (Indianapolis, Indiana, USA).

### Western blot analysis

Equal amounts of proteins were separated on SDS-PAGE, and then transferred to PVDF membranes. The membrane blots were blocked in 5% BSA for 1 h at room temperature, and then incubated with primary antibodies overnight at 4 °C. The blots were then incubated with a corresponding secondary antibody conjugated with horseradish peroxidase and signals were detected by ECL reagents and X-ray films.

### Lectin pull-down assays

To detect the Tn antigen on proteins, *Vicia villosa* agglutinin (VVA) lectin conjugated agarose beads (Vector Laboratories, Burlingame, CA) were used. Briefly, 1 mg of cell lysates were incubated with VVA agarose beads overnight at 4 °C. The agarose beads were washed several times with lysis buffer to remove any unbound proteins and then subjected to Western blot analysis.

### Flow cytometry

Cells were trypsinized and washed once with PBS. For apoptosis analysis, FITC-annexin V and propidium iodide (GeneTex) were used to stain cells for 20 min on ice and then the cells were analyzed using flow cytometer (BD Biosciences). To detect the Tn antigen on the cell surface, FITC-VVA (Vector Laboratories, Burlingame, CA) was used.

### MTT assay

PDAC cells (1.5 × 10^3^) in 100 μl medium containing 10% FBS were seeded in 96-well plates for 16 h. Ten microliters of 5 mg/ml 3-(4,5-dimethyl-2-thiazolyl)-2,5-diphenyl-2H-tetrazolium bromide solution (MTT; Sigma) was added to each well for the indicated times and incubated at 37 °C for 3 h. MTT formazan crystals were dissolved with 100 μl of 10% SDS containing 0.01 N HCl. The optical density was measured spectrophotometrically at wavelengths 550 and 630 nm.

### Transwell migration and matrigel invasion assays

Cell migration and invasion assays were performed with transwells (Corning, NY, USA) and transwells coated with Matrigel (BD Biosciences, San Jose, CA, USA), respectively. PDAC cells (5 × 10^4^) in 250 µL serum-free medium were seeded into the upper chamber and 10% FBS in the lower chamber was used as a chemoattractant. After 48 or 72 h of incubation, cells were fixed and stained with 0.5% (w/v) crystal violet (Sigma) containing 20% (v/v) methanol. The number of migrated or invaded cells from three random fields was counted under the microscope. In some experiments, 10 μg/ml of functional blocking antibodies were added to the upper chamber.

### Cell spreading analysis

PDAC cells were grown onto 100 mm culture plates. After seeding for 2 h, cells were photographed under an inverted phase-contrast microscope. Spreading cells were calculated at three different fields and are presented as mean ± SD.

### Cell adhesion assay

Ninety-six-well plates were coated with human collagen I (Sigma), human collagen IV (Sigma), human fibronectin (Sigma), murine laminin (Sigma), or bovine serum albumin (BSA) (Sigma) at concentrations of 5 μg/ml in PBS, and then blocked with 5% BSA in PBS at 37 °C for 4 h. Cells (2 × 10^4^ in 100 μl/well) in serum-free DMEM were allowed to attach for 1 h at 37 °C in a humidified 5% CO_2_ incubator. Unattached cells were washed out with PBS. Adhered cells from three wells were counted manually under an inverted microscope.

### In vivo mouse animal models

For tumor growth analysis, HPAF-II cells (5 × 10^6^) in 0.5 ml of serum-free DMEM-F12 containing 50% Matrigel (BD Biosciences) were subcutaneously injected into each NOD/SCID mouse (8–10 weeks old) with the same gender. For metastasis analysis, HPAC cells (5 × 10^6^) along with HPaSteC cells (5 × 10^6^) in 100 µl serum-free DMEM were orthotopically injected into each NOD/SCID mouse. All animal interventions were reviewed and approved by the Institutional Animal Care and Use Committee IACUC of College of Medicine, National Taiwan University.

### Statistical analysis

Statistical analyses were performed using R-4.0.2 and Prism 5 statistical software. Survival curves were plotted using the Kaplan–Meier method. Student *t* test was used to compare differences between two groups of quantitative variables. Data are presented as means ± SD or number (percentage) and *P* < 0.05 was considered statistically significant.

## Supplementary information

Supplementary Table S1

Supplementary Figures S1-S9
