# Supplementary material for: C1GALT1 high expression is associated with poor survival of patients with pancreatic ductal adenocarcinoma and promotes cell invasiveness through integrin αv
Source: Oncogene. 2021 Jan 8;40(7):1242–54. doi: 10.1038/s41388-020-01594-4 (PMC7892338; doi:10.1038/s41388-020-01594-4)
Supplement: Supplementary file 1 — Supplementary Table S1 [file 41388_2020_1594_MOESM1_ESM.docx]

**Supplementary Table S1. Demographic data and clinicopathological details of the study patients (*n* = 99).**

| **Variables** |  |
| --- | --- |
| **Age**, mean ± SD, years | 66.83 ± 12.63 |
| **Gender** |  |
| Male | 65 (65.7) |
| Female | 34 (34.3) |
| **Clinical stage†** |  |
| I | 2 (2.0) |
| II | 83 (83.8) |
| III | 10 (10.1) |
| IV | 4 (4.0) |
| **T status** |  |
| T_1-2_ | 5 (5.1) |
| T_3_ | 84 (84.8) |
| T_4_ | 10 (10.1) |
| **Nodal metastasis** | 62 (62.6) |
| **Distant metastasis** | 4 (4.0) |
| **Histologic grade** |  |
| Well differentiated | 6 (6.1) |
| Moderately differentiated | 74 (74.7) |
| Poorly differentiated | 19 (19.2) |
| **Progression** |  |
| Local recurrence | 91 (91.9) |
| Distant metastasis | 70 (70.7) |
| **Progression free survival months** |  |
| Mean ± SD | 11.64 ± 13.63 |
| Max | 90 |
| Min | 1 |
| **Overall free survival months** |  |
| Mean ± SD | 17.52 ± 17.75 |
| Max | 130 |
| Min | 1 |

Values are presented as mean ± standard deviation (SD) or number (percentage).

† TNM staging system, the AJCC 7th edition.
