# Supplementary material for: C1GALT1 high expression is associated with poor survival of patients with pancreatic ductal adenocarcinoma and promotes cell invasiveness through integrin αv
Source: Oncogene. 2021 Jan 8;40(7):1242–54. doi: 10.1038/s41388-020-01594-4 (PMC7892338; doi:10.1038/s41388-020-01594-4)
Supplement: Supplementary file 2 — Supplementary Figures S1-S9 [file 41388_2020_1594_MOESM2_ESM.pdf]

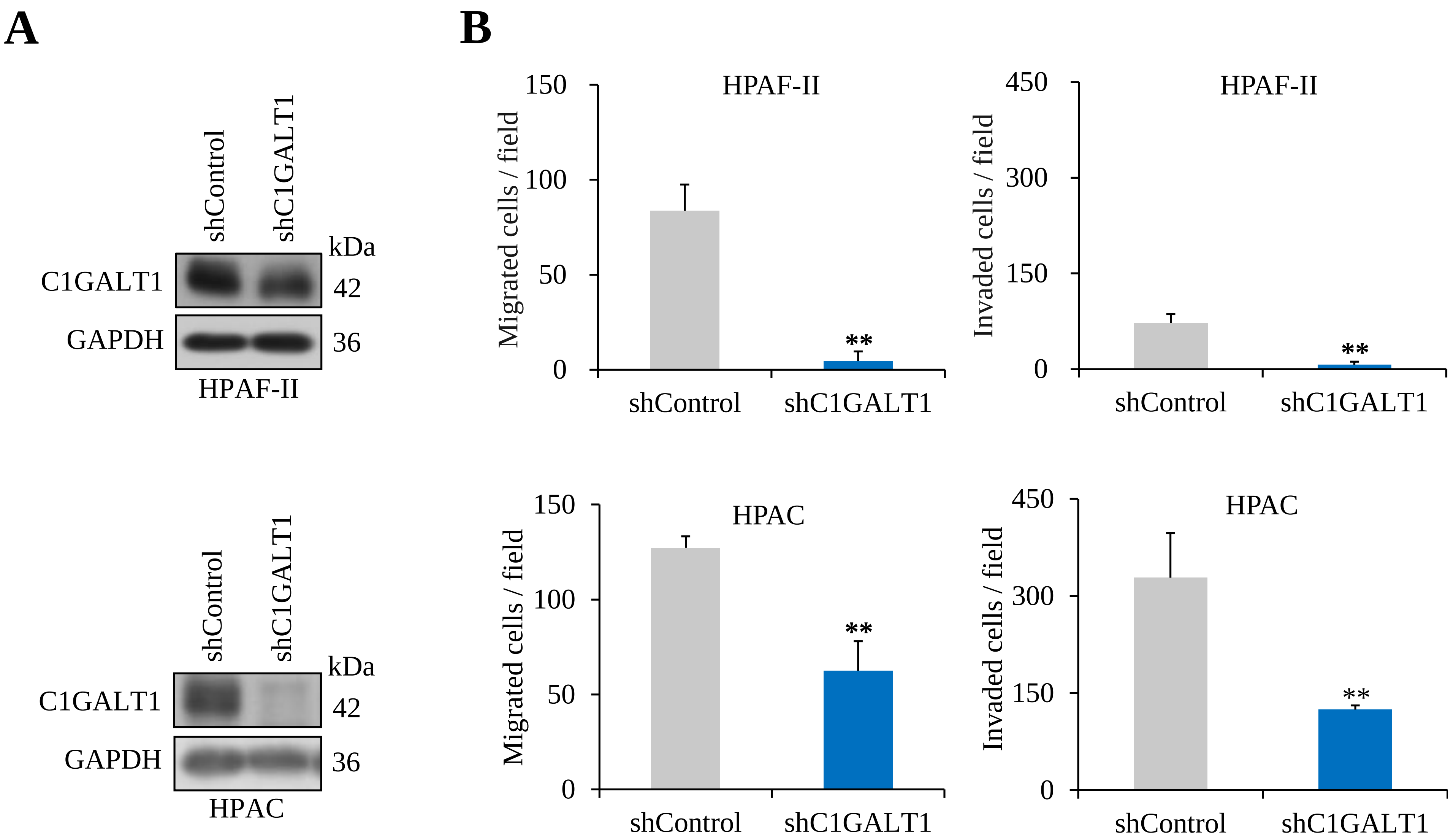

Supplementary Figure S1: C1GALT1 knockdown inhibits malignant behaviors in PDAC cells. A. Western blots showing C1GALT1 knockdown with C1GALT1 shRNA in pSUPER vector (shC1GALT1) in HPAF-II and HPAC cells. Non-targeting shRNA (shControl) was used as control. GAPDH was an internal loading control. **B.** Transwell migration and Matrigel invasion assays were performed. $n = 3$, $^{**}P < 0.01$.

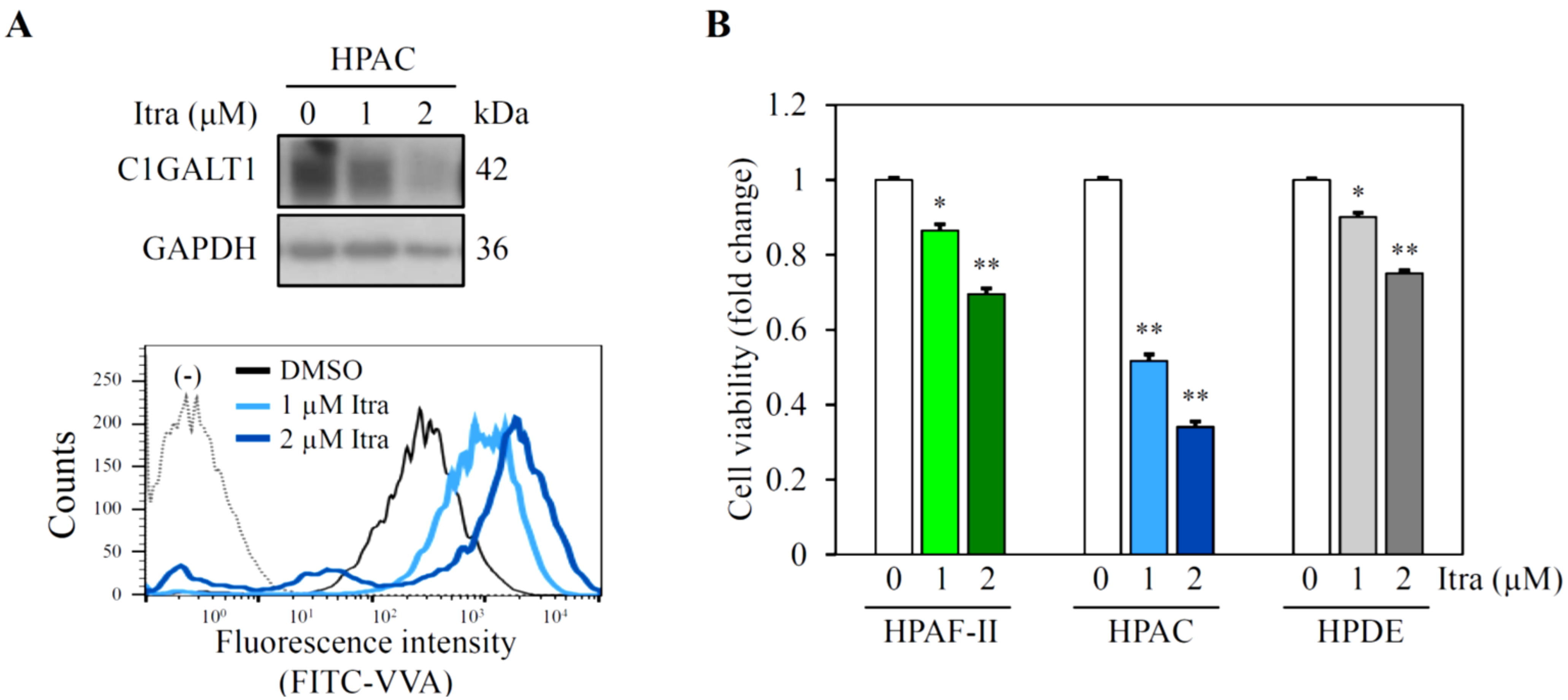

Supplementary Figure S2: Effects of C1GALT1 inhibitor itraconazole on viability of pancreatic ductal adenocarcinoma (PDAC) cells and non-transformed HPDE cells. A. Validation of itraconazole as a C1GALT1 inhibitor in PDAC cells. Upper panel, Western blots showing C1GALT1 levels in HPAC cells treated with DMSO solvent control or C1GALT1 inhibitor itraconazole (Itra) (Sigma, St. Louis, MO, USA) at different concentrations, as indicated, for 72 hours. Lower panel, changes in O-glycans on PDAC cells treated with itraconazole were confirmed using flow cytometry with FITC-VVA lectin, which recognizes Tn antigens. Non-staining control (-) is shown by a dotted line. **B.** Representative viability of cells treated with itraconazole. Cell viability of PDAC cells, including HPAF-II and HPAC cells, as well as non-transformed HPDE cells treated with different concentrations of itraconazole (Itra) for 72 hours was analyzed using MTT assays. $n = 3$. $^*P < 0.05$, $^{**}P < 0.01$.

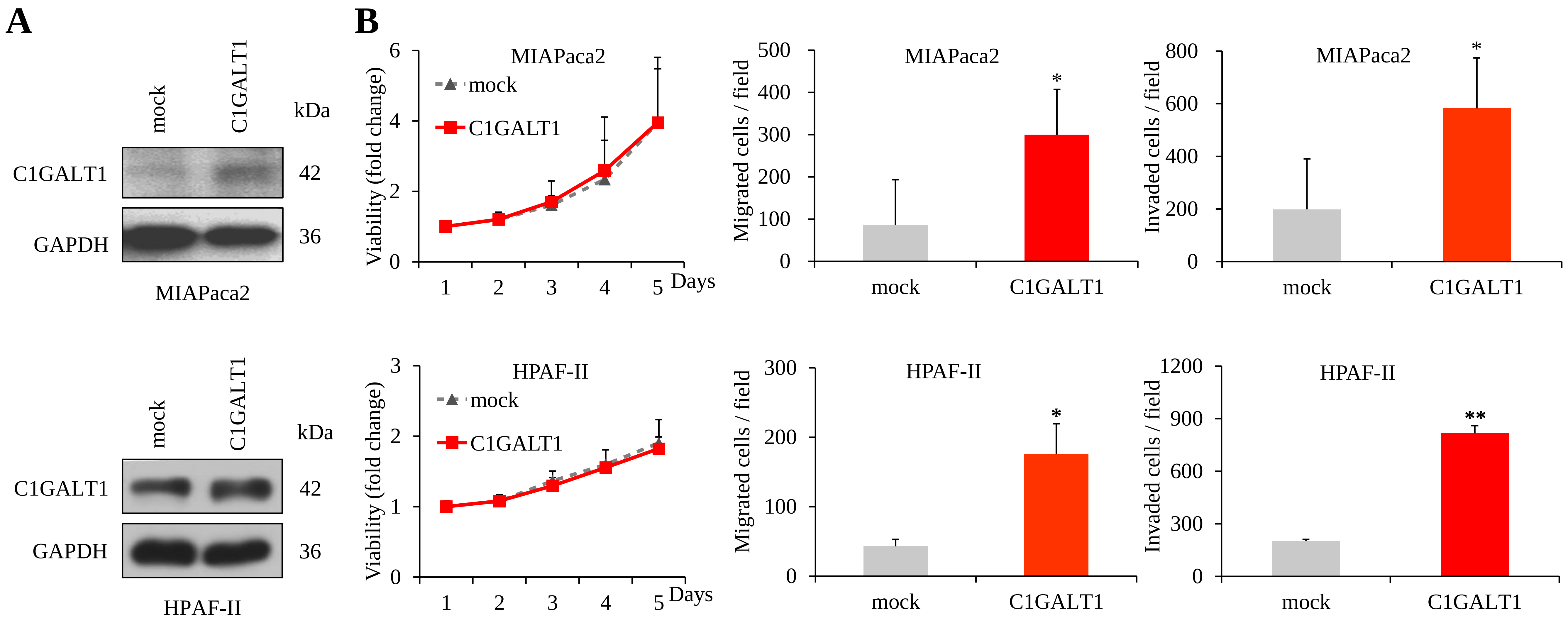

Supplementary Figure S3: C1GALT1 overexpression enhances invasive behaviors in PDAC cells. A. Western blots showing C1GALT1 overexpression using C1GALT1/pcDNA3.1 plasmid (C1GALT1) and its control empty pcDNA3.1 plasmid (mock) in MIAPaca2 and HPAF-II cells. GAPDH was used as loading control. **B.** 5-day MTT, Transwell® migration, and Matrigel™ invasion assays were performed. $n = 3$, $*P < 0.05$, $**P < 0.01$.

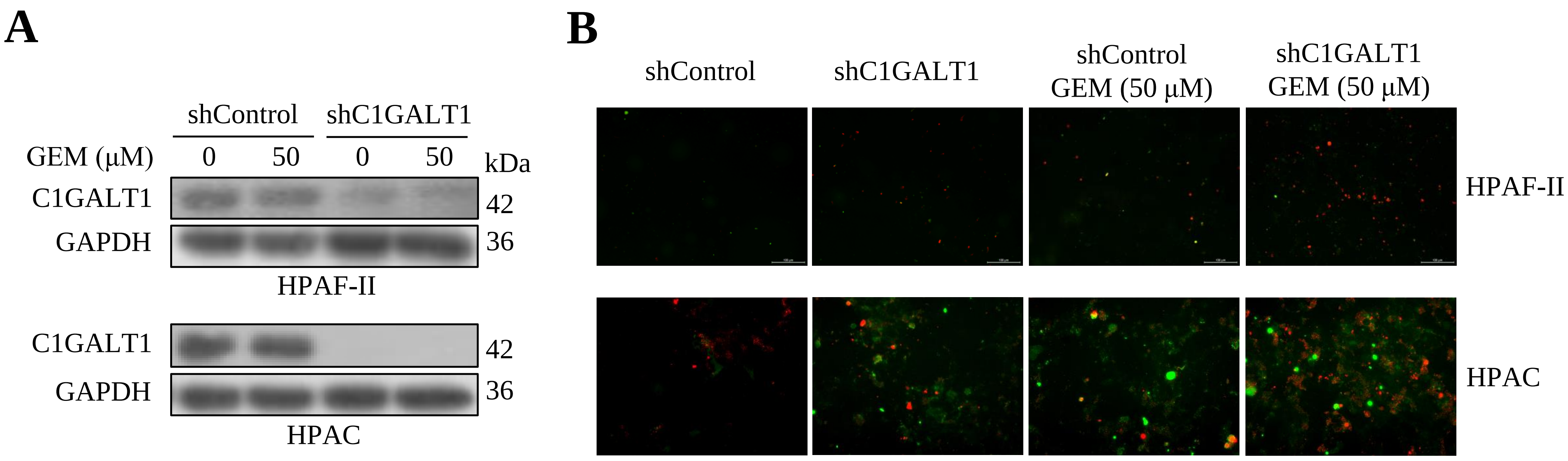

Supplementary Figure S4: C1GALT1 knockdown increases sensitivity of gemcitabine in PDAC cells. A. Western blots showing stable knockdown of C1GALT1 in HPAF-II and HPAC cells treated with or without 50 μM gemcitabine (GEM). GAPDH was used as internal control. **B.** Representative images of cells stained with FITC-annexin V (green) and propidium iodide (red) analyzed using fluorescence microscopy. Scale bar, 100 μm. Annexin V-FITC Apoptosis Detection Kit (Strong Biotech Corporation) was used to stain cells.

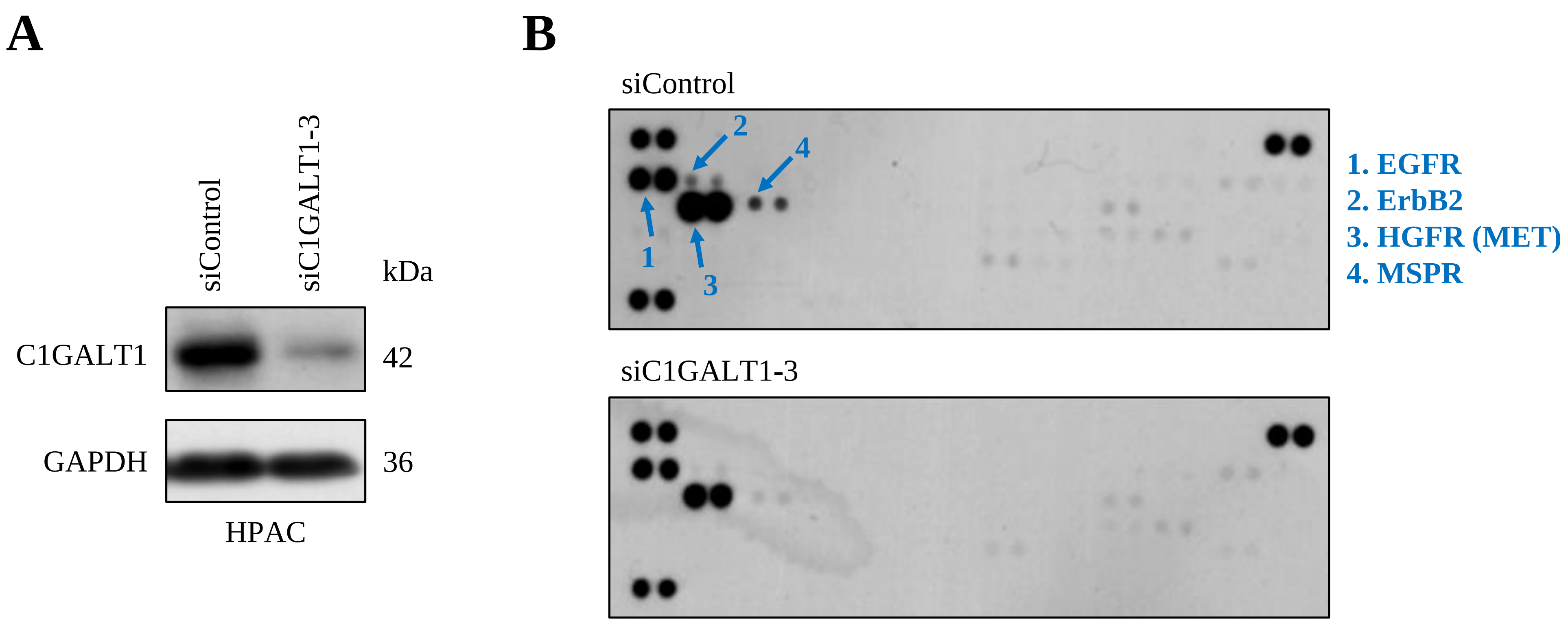

Supplementary Figure S5: C1GALT1 knockdown decreases multiple p-RTK levels. A. Western blots showing C1GALT1 knockdown with siRNA in HPAC cells. **B.** Levels of p-EGFR, p-ErbB2, p-MET, and p-MSPR, as indicated by blue arrows, were decreased in C1GALT1 knockdown cells compared with control cells. Human p-RTK Array Kit including 49 RTKs was purchased from R&D systems (Minneapolis, MN, USA). HPAC cells transfected with non-targeting siRNA (siControl) or C1GALT1 siRNA (siC1GALT1-3) were serum starved for 24 hours and then stimulated with 10% FBS for 10 minutes. Cells were lysed and 500 μg of proteins were subjected to Western blot analysis according to the manufacturer's protocol.

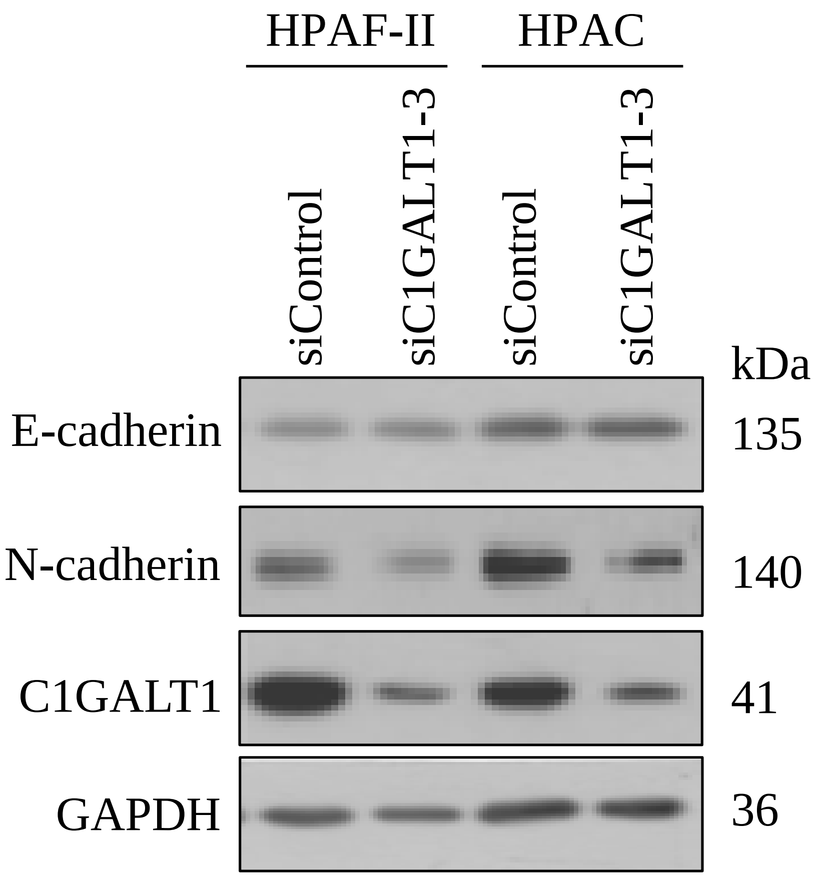

Supplementary Figure S6: C1GALT1 knockdown represses epithelial mesenchymal transition (EMT) in PDAC cells. Western blots showing effects of C1GALT1 knockdown with siC1GALT1-3 on the expression of EMT markers. Antibodies for E-cadherin and N-cadherin were purchased from Cell Signaling Technology, Inc. (Danvers, MA, USA) and BD Transduction Laboratories (San Jose, CA, USA), respectively.

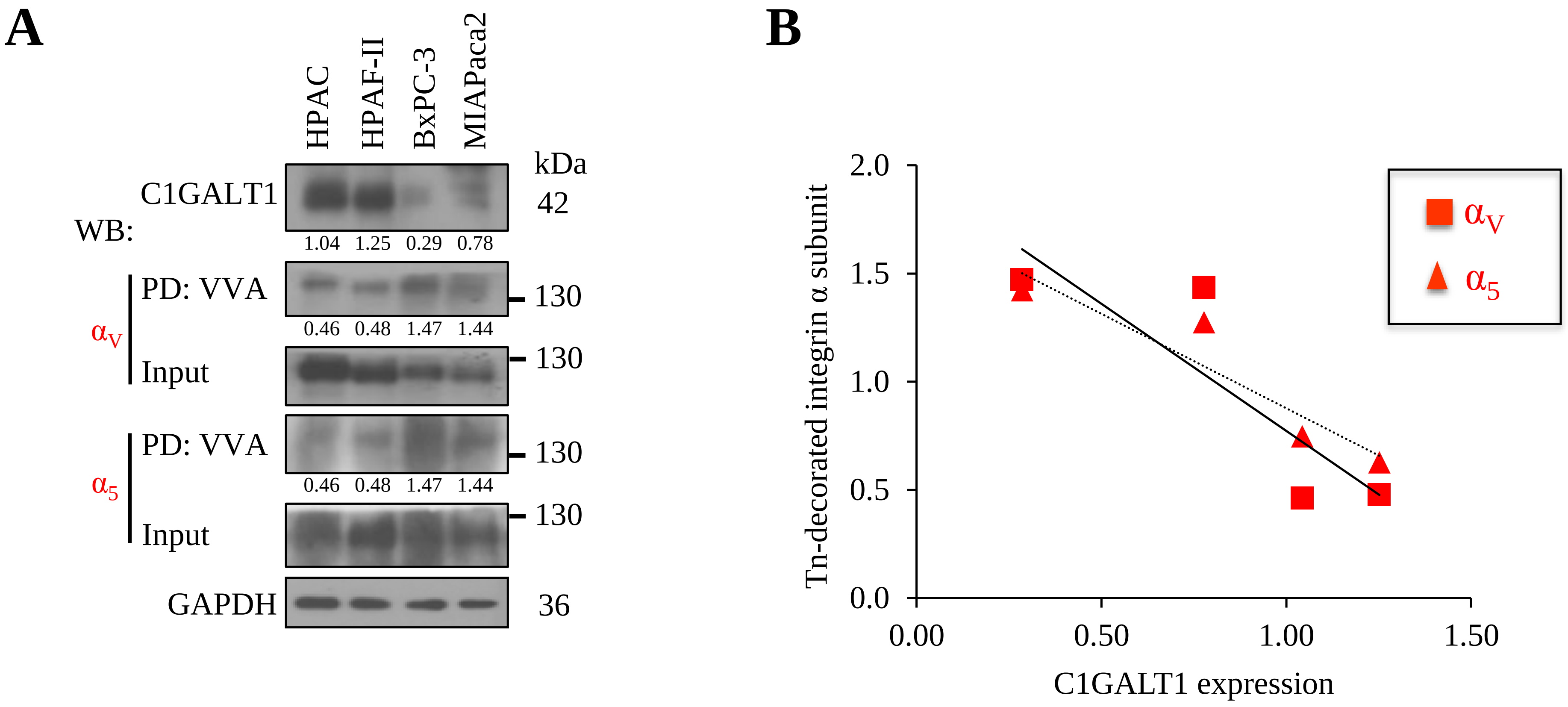

Supplementary Figure S7: C1GALT1 expression levels inversely correlate with Tn antigen levels on integrins α_V and α_5 in PDAC cells. A. Changes in Tn antigen expression on integrins were analyzed using VVA pull-down (PD) assays then Western blotted for integrins in HPAC, HPAF-II, BxPC-3, and MIAPaca2 cells. Expression of C1GALT1 and integrins in total lysates was also shown. GAPDH was used as loading control. Relative signal intensities were quantified by ImageJ and shown below each bands. Representative results from four independent experiments were shown. **B.** Inverse correlations between levels of C1GALT1 and Tn-decorated integrins α_V and α_5.

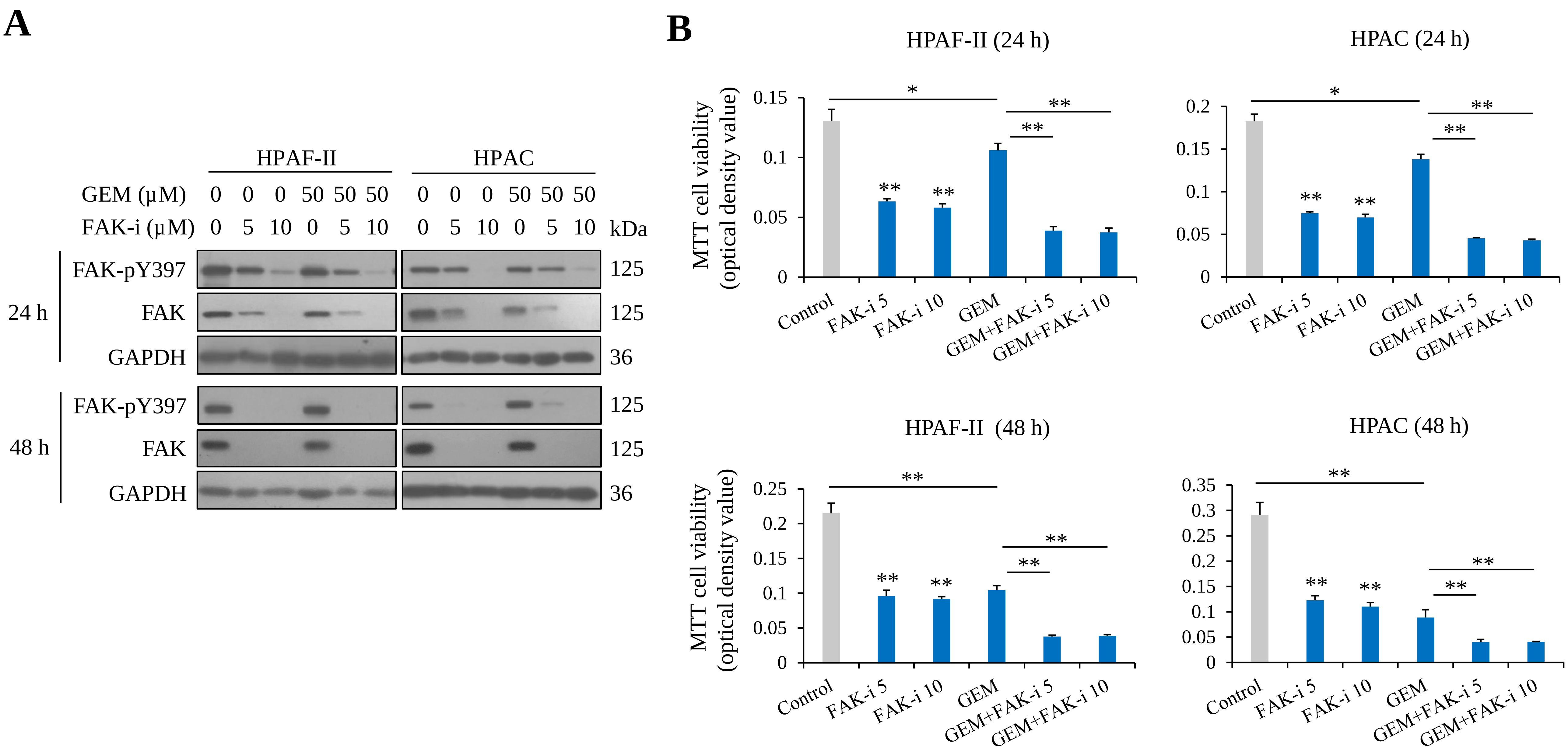

Supplementary Figure S8: FAK inhibition increases gemcitabine-induced cell death. A. Western blots showing inhibition of FAK with FAK inhibitor 14 (FAK-i) (sc-203950) purchased from Santa Cruz Biotechnology, Inc (CA, USA) in HPAF-II and HPAC cells treated with or without gemcitabine (GEM) at different concentrations, as indicated, for 24 or 48 hours (h). **B.** MTT assays. Cells were treated with 5 µM or 10 µM FAK inhibitor (FAK-i 5 or FAK-I 10) or 50 µM gemcitabine (GEM), as indicated, and then cell viability was analyzed using MTT assays. *P < 0.05, **P < 0.01.

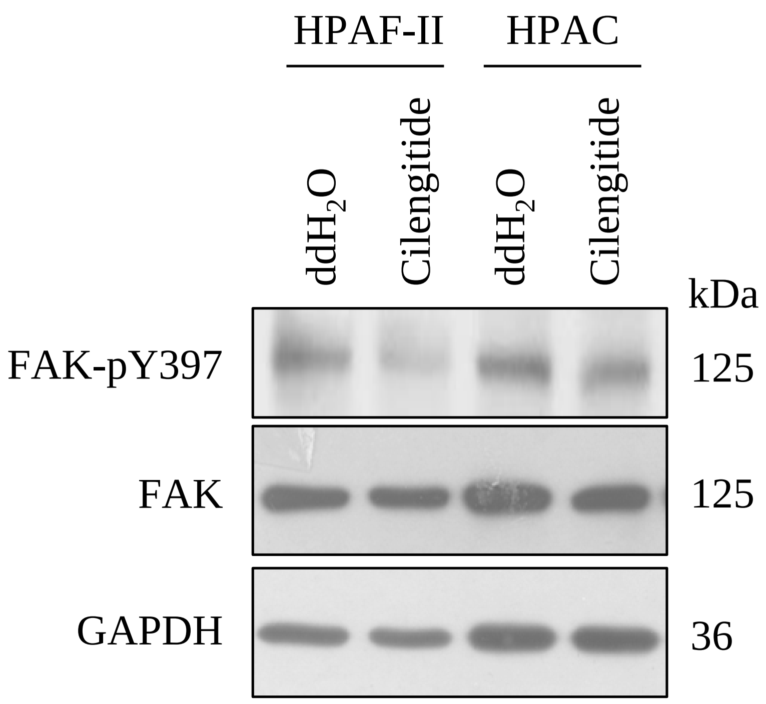

Supplementary Figure S9: Integrin inhibitor cilengitide decreased phospho-FAK levels in PDAC cells. HPAF-II and HPAC cells were treated with 5 µM cilengitide (Sigma, St. Louis, MO, USA) and FAK-pY397 levels were analyzed by Western blotting. Double distilled water (ddH_2O) was used as a solvent control.
